# A novel CircRNA Circ_0001722 regulates proliferation and invasion of osteosarcoma cells through targeting miR-204-5p/RUNX2 axis

**DOI:** 10.1007/s00432-023-05166-3

**Published:** 2023-07-15

**Authors:** Shuai Gong, Yi Zhang, Lina Pang, Liye Wang, Wei He

**Affiliations:** 1 Department of Oncology, The First Affiliated Hospital of Zhengzhou University, No. 1 of Jianshe Road, Er-Qi District, Zhengzhou City, 450052 Henan Province China; 2 Department of Orthopaedic Surgery, The First Affiliated Hospital of Zhengzhou University, Zhengzhou, 450052 Henan Province China

**Keywords:** Osteosarcoma, circRNA, miR-204-5p, Runx2, Proliferation, Invasion

## Abstract

**Background:**

Osteosarcoma (OS) is the most prevalent primary fatal bone neoplasm in adolescents and children owing to limited therapeutic methods. Circular RNAs (circRNAs) are identified as vital regulators in a variety of cancers. However, the roles of circRNAs in OS are still unclear.

**Methods:**

Firstly, we evaluate the differentially expressed circRNAs in 3 paired OS and corresponding adjacent nontumor tissue samples by circRNA microarray assay, finding a novel circRNA, circ_001722, significantly upregulated in OS tissues and cells. The circular structure of candidate circRNA was confirmed through Sanger sequencing, divergent primer PCR, and RNase R treatments. Proliferation of OS cells was evaluated in vitro and in vivo. The microRNA (miRNA) sponge mechanism of circRNAs was verified by dual-luciferase assay and RNA immunoprecipitation assay.

**Results:**

A novel circRNA, circ_001722, is significantly upregulated in OS tissues and cells. Downregulation of circ_0001722 can suppress proliferation and invasion of human OS cells in vitro and in vivo. Computational algorithms predict miR-204-5p can bind with circ_0001722 and RUNX2 mRNA 3’UTR, which is verified by Dual-luciferase assay and RNA immunoprecipitation assay. Further functional experiments show that circ_0001722 competitively binds to miR-204-5p and prevents it to decrease the level of RUNX2, which upregulates proliferation and invasion of human OS cells.

**Conclusion:**

Circ_001722 is a novel tumor promotor in OS, and promotes the progression of OS via miR-204-5p/RUNX2 axis.

**Supplementary Information:**

The online version contains supplementary material available at 10.1007/s00432-023-05166-3.

## Background

Osteosarcoma (OS) is the most prevalent primary malignant bone neoplasm causing substantial morbidity in adolescents and children (Ritter et al. 2010). It originates from mesenchymal cells and is characterized by rapid infiltrating growth, early lung metastasis and a high recurrence rate (Ottaviani et al. 2010). Approximately 80% of OS patients exhibit subclinical pulmonary micro metastases at the time of diagnosis (Jaffe 2010). Studies have shown that the overall 5-year survival rate of patients with localized OS ranges between 65 and 75% and is only 20% for those with recurrent and metastatic tumors (Miwa et al. 2019). Despite advances in OS treatment approaches such as adjuvant chemotherapy and surgical resection, the survival rates have plateaued in the last 3 decades and are less than satisfactory (Bielack et al. 2002). The lack of accurate biomarkers has further hindered efforts to improve the clinical outcome of OS. Consequently, molecular studies aiming to identify promising therapeutic targets for OS are urgently needed.

MicroRNAs (miRNAs) are a class of endogenous small RNAs with a length of approximately 20–24 nucleotides, which play various important regulatory roles in cells (Hutvágner and Zamore 2002; Meltzer 2005). Due to the ability of miRNA to regulate a large number of genes involved in processes such as proliferation, apoptosis, and DNA damage repair, abnormal expression of miRNA is closely related to tumor occurrence, development, and chemotherapy resistance (Vaghari-Tabari et al. 2020; Abolghasemi et al. 2019). Existing studies have shown that miRNAs can actively participate in the metastasis and chemosensitivity of osteosarcoma by regulating various signaling pathways(Soghli et al. 2022).Circular RNAs (circRNAs), as a type of Non-coding RNA, regulate various functions in eukaryotic cells (Meng et al. 2017). Based on the order of splicing events and different intermediates, two mechanisms exist for the biogenesis of circRNAs: canonical spliceosome induced splicing and noncanonical lariat splicing (Chen and Yang 2015; Zhou et al. 2020). Accumulating studies have shown that circRNAs modulate diverse physiological and pathophysiological processes by sponging microRNAs (miRNAs), interacting with RNA binding proteins, and modulating epigenetic, transcriptional, or translational alterations in target genes as well (Ashwal-Fluss et al. 2014; Du et al. 2016; Su et al. 2019; Li et al. 2015a). Abnormal circRNA expression has been found to correlate with the pathogenesis of various cancers and to exert essential regulatory effects on gene expression, cell invasion, cell cycle progression, migration, apoptosis, and proliferation (Wang et al. 2018; Li et al. 2018; Zhao and Shen 2017). Moreover, circRNAs are thought to possess high diagnostic and therapeutic potential given their structural stability, evolutionary conservation, abundance and organ specificity (Rybak-Wolf et al. 2015; Cui et al. 2018). However, to date, the roles of circRNAs in OS are not clearly known.

This study evaluated the expression profiles of circRNAs in OS tissues using high-throughput sequencing. We found a novel circRNA, designated circ_001722, significantly upregulated in OS tissues and cells. In addition, we found that there were two complementary pairing sites that could bind to miR-204-5p on the circ_001722. MiR-204-5p was initially discovered during the development of posterior capsule opacification in humans, and its role is to regulate the transition of epithelium to mesenchymal tissue, which is a key process in cancer cell metastasis (Wang et al. 2013). Later, Researchers found that miR-204-5p was involved in the migration and/or invasion of endometrial cancer cells, colorectal cancer cells, gliomas, oral squamous cell carcinoma, and laryngeal squamous cell carcinoma (Bao et al. 2013; Yin et al. 2014; Xia et al. 2015; Wang et al. 2016; Gao et al. 2017). These evidences indicate that miR-204-5p plays an anticancer role in various types of cancer by inhibiting metastasis. Subsequently, we identified Runx2 as a possible target gene for miR-204-5p. RUNX proteins are DNA binding transcription factors that regulate the expression of multiple genes involved in cell differentiation and cell cycle progression (Kagoshima et al. 2007). Runx2, as a member of the RUNX family, is highly expressed in bone tissue and is a highly active molecule in the development, differentiation, and maturation of bone tissue, as well as the occurrence and development of various tumors (Li et al. 2012).Some studies have shown that the RUNX2 DNA copy number, RNA and protein levels in Osteosarcoma are highly elevated (Martin et al. 2011). The protein also plays an important role in bone metastasis of prostate cancer and breast cancer (Ito et al. 2015; Kanwal et al. 2018). These studies suggest that RUNX2 has a relevant impact on the development of bone Morphogenesis and Osteosarcoma. Based on our previous work and current research status, we consider that the abnormally elevated expression of circ_0001722 in Osteosarcoma cells may participate in the proliferation and invasion of Osteosarcoma by affecting the expression of miR-204-5p and Runx2. Afterwards, we further validated their relationship and possible mechanisms of mutual influence.

## Materials and methods

### Patients and OS samples

A series of 20 surgically resected fresh human OS and corresponding adjacent nontumor tissue samples were collected at the First Affiliated Hospital of Zhengzhou University (Zhengzhou, China) and snap-frozen in liquid nitrogen from July 2018 to January 2019. Among them, 3 pairs were used for circRNA microarray analysis. No patients had received any preoperative treatment. Clinical data of patients included in this study are detailed in Supplementary Table 1. Samples used in this study were approved by the Committees for Ethical Review of the First Affiliated Hospital of Zhengzhou University.

### CircRNA microarray analysis

Three pairs of human OS and corresponding adjacent nontumor tissue samples were used for the circRNA microarray assay to determine differentially expressed circRNAs. The microarray hybridization was performed based on the manufacturer’s standard protocols (Agilent Technologies, USA), which included purifying the RNA, transcribing it into fluorescent cRNA, and then hybridizing it onto the Human circRNA Arrays (Agilent Technologies, USA). Finally, the hybridized slides were washed, fixed and scanned to images by an Agilent Scanner G2505C. The data collection was performed using Agilent Feature Extraction software (version 11.0.1.1). The raw data were quantile normalized, and further data analysis was performed with the *R* software package, GeneSpring GX (Agilent Technologies, USA) and gene expression dynamics inspector (GEDI). The statistical significance of differentially regulated circRNAs between OS tissue (T) and adjacent nontumor tissue (N) was identified through p-values and fold changes. Significantly differentially expressed transcripts were retained by screening for a fold change ≥ 2.0 and *P* < 0.05. Hierarchical clustering was performed to generate an overview of the characteristics of expression profiles based on the values of all expressed transcripts and significant differentially expressed transcripts.

### Circular structure confirmation

The circular structure of circ_0001722 was confirmed by RNase R treatment and Sanger sequencing by divergent primer PCR. For RNase R treatment, 3 μg total RNA extracted from OS tissues and cell lines were incubated with 20 U RNAse R (Epicentre Biotechnologies, USA) in a 10 μl volume at 37 °C for 45 min, followed by 70 °C for 10 min to deactivate the RNase R. The treated RNAs were used for RT-PCR. For Sanger sequencing, PCR products amplified by divergent primers of circ_0001722 were inserted into the T vector and sequenced by Tsingke Biotechnology (Beijing) Co., Ltd. The result was crosschecked with the back-splicing junction sites of circ_0001722 supplied by circBASE (Chen et al. 2019).

### Cell culture, transfection, and lentiviral infection

Two human OS cell lines (MG63 and U2OS), a normal human fetal osteoblastic cell line (hFOB1.19) and a human embryonic kidney cell line (HEK293T) used in this study were purchased from Chinese National Collection of Authenticated Cell Cultures. MG63 cells were cultured in DMEM medium (Hyclone, USA) supplemented with 10% fetal bovine serum (HyClone, USA) and 1% penicillin/streptomycin (Invitrogen, USA) at 37 °C under 5% CO_2_ and saturated moisture. U2OS cells were cultured in McCoy's 5a medium (Hyclone, USA) supplemented with 10% fetal bovine serum (HyClone, USA) and 1% penicillin/streptomycin (Invitrogen, USA) at 37 °C under 5% CO_2_ and saturated moisture. hFOB1.19 cells were cultured in DMEM/F12 (1:1) medium (Hyclone, USA) supplemented with 10% fetal bovine serum (HyClone, USA) and 1% penicillin/streptomycin (Invitrogen, USA) at 37 °C under 5% CO_2_ and saturated moisture. HEK293T cells were cultured in DMEM/high glucose medium (Hyclone, USA) supplemented with 10% fetal bovine serum (HyClone, USA) and 1% penicillin/streptomycin (Invitrogen, USA) at 37 °C under 5% CO_2_ and saturated moisture. The authenticity of cell lines was verified by DNA fingerprinting before use. MiR-204-5p and its negative control (miR-NC), RUNX2 eukaryotic expression recombinant pIRESpuro2-RUNX2 were transfected transiently into OS cells using Lipofectamine 3000 (Invitrogen, USA) according to the manufacturer’s instructions. To prepare sh-circ_0001722 lentiviral particles, the lentiviral vector harboring sh-circ_0001722 full hairpin sequence and packaging vectors were transfected into HEK293T cells using iMFectin Poly DNA Transfection Reagent (GenDEPOT, USA) following the manufacturer’s suggested protocols. The transfection medium was changed at 8 h after transfection and then cells were cultured for 36 h. The lentiviral particles were harvested by filtration using a 0.45 µm sodium acetate syringe filter and then combined with 8 μg/ml of polybrane (Millipore, USA) and infected overnight into 60% confluent OS cells. The cell culture medium was replaced with fresh complete growth medium and after 24 h, cells were selected with 2 μg/ml of puromycine for an additional 24 h. The selected cells were used for experiments. MiR-204-5p mimic and its negative control (miR-NC), pIRESpuro2-RUNX2 vectors, lentiviral vectors harboring sh-circ_0001722 full hairpin sequence and mock sequence, were purchased from Shanghai GenePharma Co., Ltd.

### RNA extraction and qRT-PCR analysis

Total RNA derived from human OS tissues and cells was isolated using TRIzol reagent (TAKARA, CHN) according to the manufacturer’s instructions. RNA was reverse transcribed into cDNA using a Primer-Script one step RT-PCR kit (TAKARA, CHN). Quantitative real-time PCR experiments were performed using a SYBR Premix Dimmer Eraser kit (TAKARA, CHN) on an ABI 7500 Real-Time PCR System (Applied Biosystems, USA). The fold change in relative expression level was calculated using the 2^−ΔΔCt^ method. Relative circ_0001722 expression was normalized to GAPDH expression, and miR-204-5p expression was normalized to U6 small nuclear RNA (U6 snRNA). The primer sequences used in our study are purchased from Tsingke Biotechnology (Beijing) Co., Ltd and shown in Supplementary Table 2.

### Cell proliferation assay

The proliferation of human OS cells was evaluated by the CellTiter 96 AQueous One Solution cell proliferation assay. human OS cells were plated in 96-well culture plates (3 × 10^3^ per well). After 24 h of incubation, the cells were transfected with 30 pmol of target gene (sh-circ_0001722 or miR-204-5p or negative control) for 24, 48, 72, and 96 h. Then 20 μl Cell Titer 96 Aqueous One Solution (Promega, USA) were added and cells incubated for another 1 h. Absorbance was read at 492 nm.

### Matrigel invasion assay

The invasion abilities of OS cells were evaluated using Transwell invasion chambers precoated with 50 μl of 2 mg/ml Matrigel (BD Biosciences, USA). In brief, 5 × 10^4^ transfected cells suspended in 200 μl of serum-free DMEM were seeded into the upper chambers. A 600 μl volume of DMEM supplemented with 10% FBS was used as the attractant and was added into the lower chambers. After culture for 24 h, cells adhering to the lower surface of the membrane were fixed with paraformaldehyde (4%) and stained using crystal violet (0.1%), whereas cells on the upper surface of the membrane were removed by wiping with cotton swabs. At least three random fields of view containing cells that had migrated or invaded to the lower surface were imaged under an inverted light microscope.

### Dual-luciferase assay

HEK293T cells were spread to 96 well plates at the concentration of 1 × 10^4^ cells per well. After 24 h, HEK293T cells were co-transfected with dual-luciferase reporter vector (pmirGLO-Wt-circ or pmirGLO-Mt-circ, pmirGLO-Wt-3’UTR or pmirGLO-Mt-3’UTR) and miR-204-5p mimics or negative control (miR-NC) using the Lipo-fectomine 3000 transfection reagent (Invitrogen, USA), respectively. After 48 h of incubation, firefly and Renilla luciferase activities were measured using a dual-luciferase reporter assay system (Promega, USA) according to the manufacturer’s instructions.

### RNA immunoprecipitation (RIP) assay

The RIP assay was performed using a Magna RIP RNA Binding Protein Immunoprecipitation Kit (Bersinbio, China) according to the manufacturer’s protocol. 2 × 10^7^ MG63 or U2OS cells were lysed in complete RIP lysis buffer and the cell lysates were divided into two equal parts and incubated with either 5 μg human anti-Argonaute2 (AGO2) antibody (Millipore, USA) with rotation at 4 °C overnight. Magnetic beads were added to the cell lysates and incubation was continued at 4 °C for 1 h. The samples were then incubated with Proteinase K at 55 °C for 1 h. The enriched RNA was obtained using RNA Extraction Reagent (Solarbio, CHN). The purified RNA was used to detect the expression levels of the genes of interest by qRT-PCR.

### Western blot analysis

Cells collected from different treatment groups were added to RIPA buffer (Beyotime Biotechnology, Nantong, China) containing 1% protease inhibitor (cell signaling technology) for lysis. Protein concentrations of cell lysates were determined using a protein assay kit (Bio-Rad Laboratories, USA). Total proteins (20 μg) were loaded and separated on an 8% sodium dodecyl sulfate–polyacrylamide gradient gel. The proteins were then transferred onto a polyvinylidene difluoride membrane (Millipore, USA). After blocking in 5% non-fat milk, the membranes were probed with primary antibodies (RUNX2: 1:500, sc-101145, GAPDH: 1:2000, sc-47724, Santa Cruz Biotechnology, USA) overnight at 4 °C, then washed 3 times with TBS-Tween 20 followed by incubation at room temperature 1 h with a horseradish peroxidase (HRP)-conjugated secondary antibody. The protein bands were visualized with an Immobilon Western Chemiluminescent HRP Substrate (Millipore, USA).

### Xenograft nude mouse model

Six-week-old male BALB/C nude mice (Vital River Laboratory Animal Technology, Beijing, CHN) were maintained under specific pathogen-free conditions with a 12 h light/dark cycle. All animal experiments were performed in accordance with the guide lines for the Care and Use of Laboratory Animals of Zhengzhou University. MG63 cells stably transfected with sh-circ_0001722 lentivirus or control lentivirus were subcutaneously injected into the right upper back of the nude mice (5 × 10^6^ cells per mouse). Four weeks later, the mice were sacrificed and tumor tissues were collected for examination of the parameters of interest.

### Immunohistochemistry staining

Immunohistochemical analysis for Ki67 was performed on 4-μm sections. The Envision Plus detection system (Dako, USA) was used for the detection of immunostaining. Tissue sections were pretreated with 10 mM sodium citrate buffer for antigen unmasking (pH 6.0) after deparaffinized in xylene. Endogenous peroxidase activity was blocked by incubation with 0.03% hydrogen peroxide in methanol for 15 min. Then sections were incubated with Ki67 primary antibody (MA5-14,520, Thermo Scientific, USA) at 4 °C overnight after blocked in normal serum for 30 min. Next, Sections were incubated with secondary antibody at room temperature for 60 min before staining for 5 min with 3′3-diaminobenzidine tetrahydrochloride, counterstained by hematoxylin, and observed by microscope (200 ×).

### Statistical analysis

Data for continuous variables are presented as means ± standard deviations. All analyses were performed using SPSS 21.0 software (IBM, USA). All experiments were performed with three technical replicates, and at least three biological replicates were performed. Differences between groups were analyzed using unpaired Student’s t-test or one-way analysis of variance (ANOVA) with Tukey’s test. A *P* value of < 0.05 was considered to be statistically significant.

## Results

### The results of circRNA identification and annotation

The reads were aligned to the reference genome using bwa mem software. The read distribution of OS and corresponding adjacent nontumor tissues on the chromosomes were shown in Supplementary Fig. 1a, b, separately. After bwa mem alignment, sam files were subjected to CIRI2 processing twice. Firstly, the junction reads were detected by paired chiastic clipping (PCC) signals. Basing on paired-end mapping (PEM) and GT-AG sequence feature, the reads were preliminary filtered to obtain candidate circRNAs. Secondly, the junction reads were detected again to filter out the false-positive candidate circRNAs (Supplementary Fig. 1c). Totally, 6646 circRNAs were identified in OS and nontumor tissues, and in each type of samples, with 1–3 circRNAs on most genes. Among these 6646 circRNAs, 6014 circRNAs were distributed on exon region, 459 circRNAs were distributed on intron region, and the rest 173 circRNAs were distributed on intergenic region. Moreover, 4275 circRNAs have been annotated before, and the remaining 2371 were newly annotated. The lengths of 6646 circRNAs were mainly distributed in the range of 150–1000 bp (Supplementary Fig. 1d).

### Differentially expressed circRNAs and circRNA/miRNA/mRNA regulatory network

**Fig. 1:**
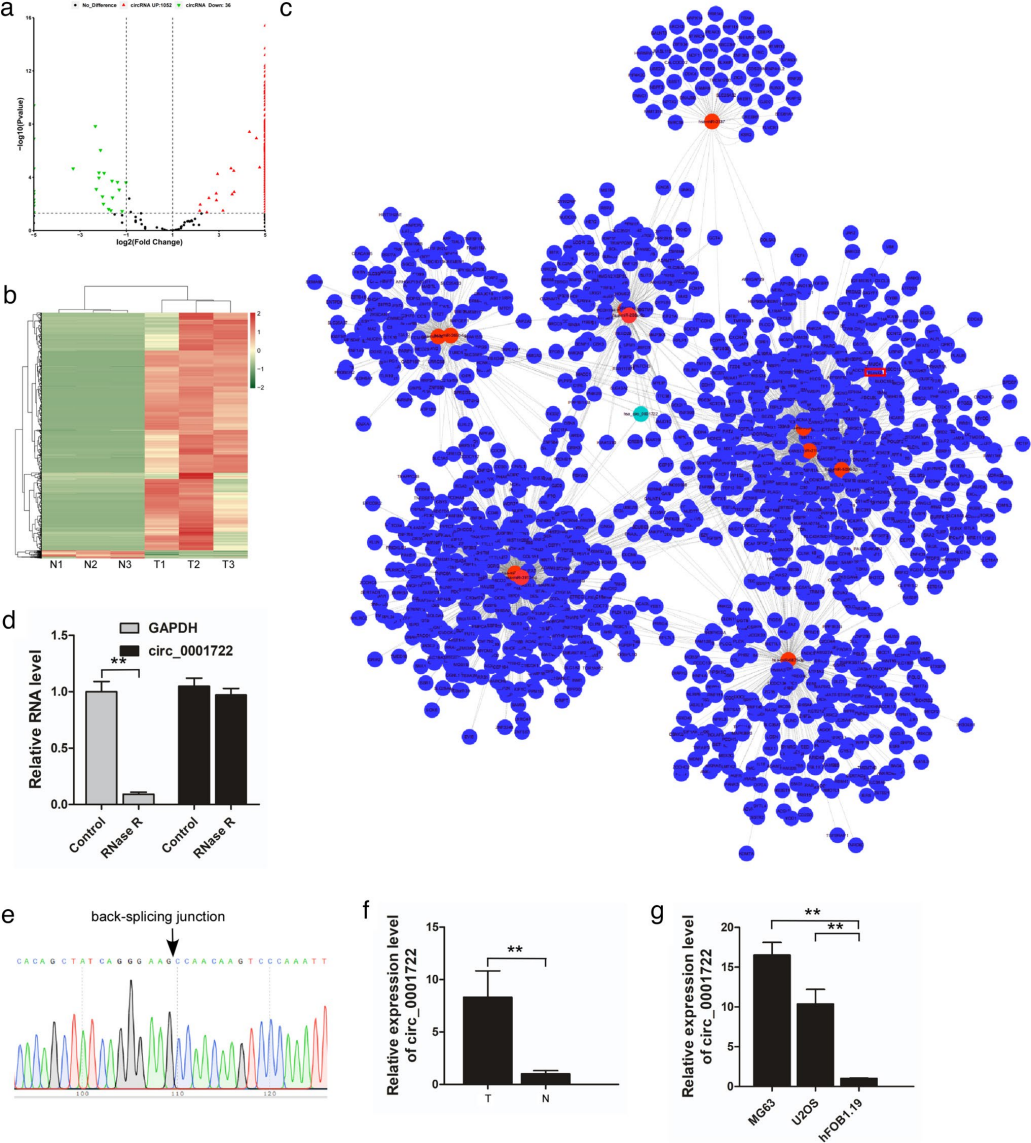
Identification and validation of differential expression of circ_0001722 in OS tissues and corresponding adjacent nontumor tissues. **a** CircRNA expression profiling between two groups is showed with volcano plot. The vertical lines refer to a 2.0-fold (log2 scaled) up-regulation and down-regulation, respectively. The horizontal line corresponds to a *P* value of 0.05 (− log10 scaled). The red points represent up regulated circRNAs with statistical significance, and green points represent down regulated ones. **b** Hierarchical clustering heatmap indicates differences in circRNA expression profiling between the two groups. **c** 11 target miRNAs of circ_0001722 are predicted by miRanda and TargetScan, as well as their corresponding target cancer related mRNA, estimated by miRTarBase database. Green: circ-0001722; red: miRNAs; blue: mRNAs. **d** Relative levels of circ_0001722 and GAPDH in OS cell line MG63 after their RNAs are treated with or without RNase R digestion. **e** The validation of back-splicing junction sites of circ_0001722 by Sanger sequencing. **f** The relative expression of circ_0001722 was detected in 20 OS tissues and corresponding adjacent nontumor tissues. **b** The relative expression of circ_0001722 was detected in two OS cell lines and an immortalized human fetal osteoblastic cell line. **P < 0.01

Additionally, we have also identified the differentially expressed circRNAs between OS and corresponding adjacent nontumor tissue samples. Compared with nontumor samples, there were totally 1088 significantly differentially expressed circRNAs in OS samples, including 1052 upregulated circRNAs and 36 downregulated circRNAs (Fig. 1a). The expression levels of differentially expressed circRNAs were significantly different (Fig. 1b).

Furthermore, we have estimated the circRNA/miRNA interactions using miRanda and TargetScan. After the cross analysis of predicted results of two software, there were 36 differentially expressed circRNAs and their corresponding miRNAs in regulatory network (Supplementary Fig. 2a). Among them, circ_0001722 with small expression standard deviations in three OS samples and three nontumor samples (standard deviation values were 3.1 and 0.7, respectively) was selected for subsequent analysis. Regarding circ_0001722, there were 11 overlapped regulatory pairs between miRanda and TargetScan (Supplementary Fig. 2b), including miR-365b-5p, miR-3122, miR-211-5p, miR-208b-5p, miR-3913-5p, miR-204-5p, miR-365a-5p, miR-5006-3p, miR-208a-5p, miR-3137 and miR-6875-3p. Then, the target genes of these 11 miRNAs were also estimated utilizing miRTarBase database. We found that miR-204-5p and its corresponding 76 target genes, comprising RUNX2, were supported by most experimental validation (469 experimental results). The circRNA/miRNA/mRNA regulatory network, based on circ_0001722, 11 miRNAs, and target mRNAs, was shown in Fig. 1c.

### Expression of circ_0001722 is significantly upregulated in OS tissues and cell lines

Firstly, we determined whether circ_0001722 is a closed circular RNA that is resistant to RNase R digestion. We investigated its expression level in OS tissues and cell lines. Our result showed that RNase R digestion could decrease the RNA level of linear GAPDH, but could not affect the level of circ_0001722 significantly (Fig. 1d), indicating that circ_0001722 was resistant to RNase R digestion. To confirm circ_0001722 is a closed circRNA, we ran PCR amplification of circ_0001722 with specific divergent primers. PCR products amplified by divergent primers were detected by Sanger sequencing to confirm the circular structure of circ_0001722. Our data showed that the back splicing junction sites of circ_0001722 were consistent with the sequence from circBase (Fig. 1e). Then, we measured the expression level of circ_0001722 in a series of 20 surgically removed fresh OS tissues and their corresponding adjacent nontumor tissues by qRT-PCR. Comparing with nontumor tissues, the expression level of circ_0001722 in OS tissues was significantly upregulated (*P* < 0.01; Fig. 1f). Similarly, the expression of circ_0001722 in OS cell lines (MG63 and U2OS) was significantly higher than in normal human fetal osteoblastic cell line (hFOB 1.19) (*P* < 0.01; Fig. 1g).

### Downregulation of circ_0001722 suppresses cell proliferation of human OS cells in vitro and in vivo.

**Fig. 2:**
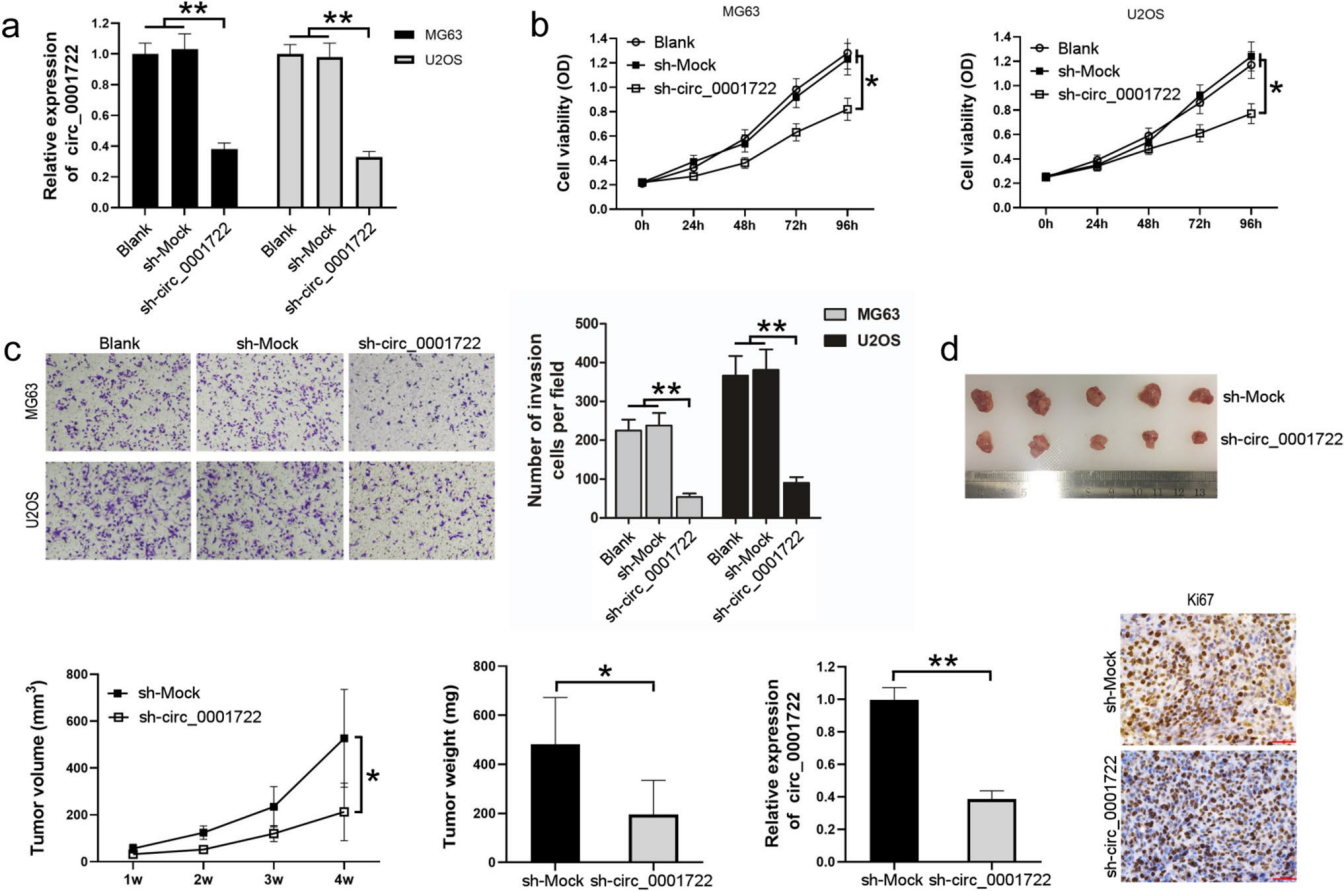
Downregulation of circ_0001722 suppresses cell proliferation of human OS cells. **a** circ_0001722 is down-regulated by exogenous shRNA (sh-circ_0001722) in two human OS cell lines. **b** Down-regulation of circ_0001722 can suppress cell proliferation of both human OS cell lines in vitro. **c** Downregulation of circ_0001722 can suppress the invasion ability of both human OS cell lines. **d** Downregulation of circ_0001722 can suppress cell proliferation of MG63 cells in vivo. *P < 0.05. **P < 0.01

We noticed that the expression of circ_0001722 was higher in MG63 cells and U2OS cells. Based on the observation, exogenous shRNA (sh-circ_0001722) was used to knock down circ_0001722 expression in two OS cell lines (Fig. 2a). Based on the high silencing efficiency, we carried out cell proliferation assay and matrigel invasion assay. The results showed that the viability level of MG63 cells and U2OS cells transfected with sh-circ_0001722 was lower than that of the cells transfected with sh-Mock (Fig. 2b). Consistently, sh-circ_0001722 significantly decreased the number of OS cells invaded through the matrigel. Quantitative analysis of cell numbers revealed that, in the negative control (sh-Mock) and blank control groups, the number of cells invaded through the matrigel was almost 5 times higher than sh-circ_0001722 group (Fig. 2c). The above experiments verified to some extent that silencing circ_0001722 can inhibit the proliferation and invasion of OS cells in vitro. To investigate whether circ_0001722 regulates the tumorigenesis of OS in vivo, we established OS xenograft mouse models. MG63 cells transfected with sh-circ_0001722 or sh-Mock were inoculated subcutaneously in the right flank of athymic nude mice (5 × 10^6^ cells per mouse, 5 mice per group). After 4 weeks, all experimental mice were euthanized and tumor tissues were collected (Fig. 2d). Comparing with sh-Mock group, smaller tumor volume and lower tumor weight were observed in sh-circ_0001722 group. The same conclusion was reached with the in vitro experimental results. Downregulation of circ_0001722 can suppress tumorigenesis of OS cells in vivo. In addition, compare with the control group, knocking down of circ_0001722 led to the decrease of Ki67 expression, which implied the cell proliferation of sh-circ_0001722 group is slower than sh-Mock group. Taken altogether, downregulation of circ_0001722 can restrain the growth of OS cells in vitro and in vivo.

### Circ_0001722 acts as a sponge for miR-204-5p.

**Fig. 3:**
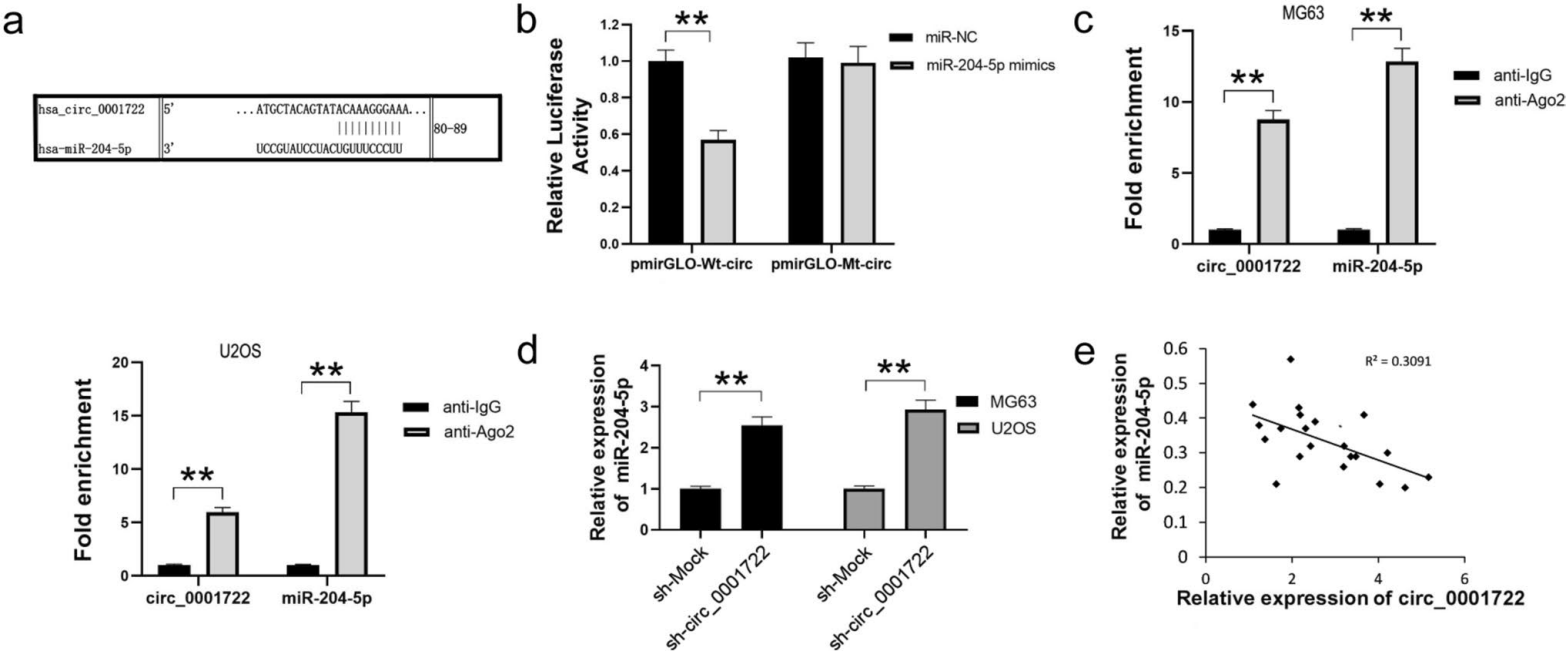
circ_0001722 binds to miR-204-5p to suppress its expression in human OS cells. **a** Computational algorithms predicts complementary sequences of the circ_0001722 and miR-204-5p binding sequence. **b** Compared with miR-NC, miR-204-5p mimic can significantly reduce the luciferase activity of pmirGLO-Wt-circ clone in HEK293T cells. However, neither miR-NC nor miR-204-5p mimic can affect the luciferase activity of pmirGLO-Mt-circ clone. **c** Ago2 RIP assay shows that Ago2 can significantly enrich circ_0001722 and miR-204-5p. **d** Downregulation of circ_0001722 can promote miR-204-5p expression in both human OS cell lines. e: The expression of miR-204-5p was negatively associated with circ_0001722 in human OS tissues. NC: negative control. Wt: wild type. Mt: mutant type. **P < 0.01

The sequences of circRNAs are highly conservative, and circRNAs can function as miRNA sponge to regulate gene expression. Through bioinformatical analysis, we discovered that complementary pairing sites (80–89 nt) on circ_0001722 that could bind to miR-204-5p (Fig. 3a). To validate binding capability of the miR-204-5p to circ_0001722, we constructed the circ_0001722 luciferase reporter system. In the dual-luciferase reporter assay, comparing with miR-NC, miR-204-5p mimics could significantly suppressed the luciferase activity of pmirGLO-Wt-circ in HEK293T cells, while the luciferase activity of pmirGLO-Mt-circ could not be suppressed (Fig. 3b). We next performed Ago2 immunoprecipitation to determine whether circ_0001722 served as a platform for Ago2 and miR-204-5p. As shown in Fig. 3c, circ_0001722 and miR-204-5p were abundantly enriched more in Ago2 protein than in IgG, suggesting that expression of miR-204-5p could be affected by circ_0001722. Moreover, the expression of miR-204-5p was elevated in circ_0001722 silenced MG63 and U2OS cells (Fig. 3d). Furthermore, the results of Pearson’s correlation analysis showed the circ_0001722 level was inversely to correlate with miR-204-5p level in 20 human OS tissues (Fig. 3e), which providing evidence of the potential correlation between circ_0001722 and miR-204-5p. Given all of these data, circ_0001722 not only targeted miR-204-5p but also acted as a sponge for miR-204-5p in OS cells.

### Sh-circ_0001722 inhibits the proliferation and invasion of human OS cells by upregulating miR-204-5p

**Fig. 4:**
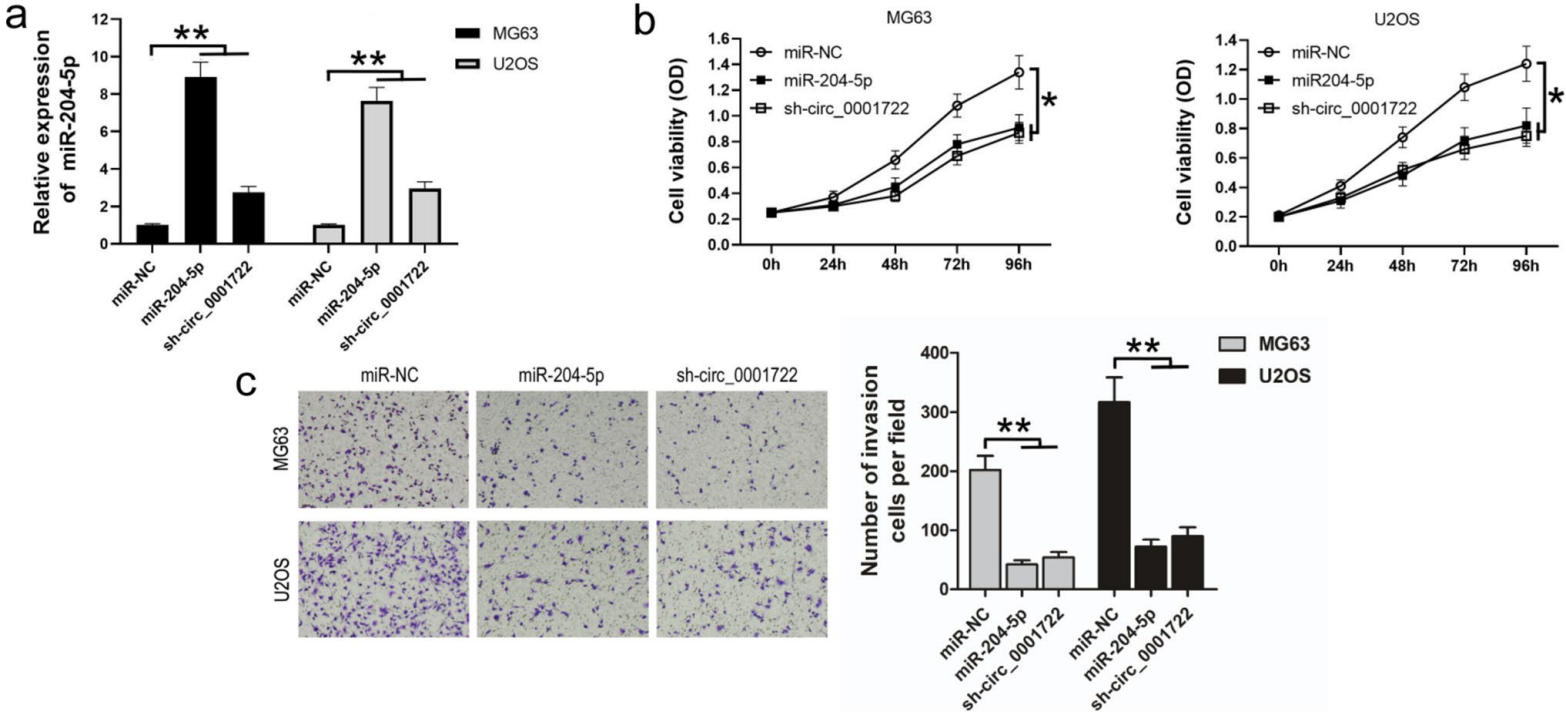
Sh-circ_0001722 suppresses proliferation and invasion of human OS cells by upregulating miR-204-5p. **a** Both miR-204-5p and sh-circ_0001722 can upregulate miR-204-5p expression level in two human OS cell lines. **b** Both miR-204-5p and sh-circ_0001722 can suppress cell proliferation of human OS cell lines in vitro. **c** Both miR-204-5p and sh-circ_0001722 can suppress the invasion ability of human OS cell lines in vitro. NC: negative control. *P < 0.05. **P < 0.01

In order to further investigate whether circ_0001722 plays a promotion role in OS cells through binding with miR-204-5p, we conducted the following experiments. We transfected MG63 and U2OS cells with miR-204-5p, making miR-204-5p expression increased remarkably, which also could be observed after sh-circ_0001722 transfection as well (Fig. 4a). The cell proliferation assays showed that both upregulation of miR-204-5p and downregulation of circ_0001722 could suppress OS cell proliferation ability in vitro (Fig. 4b). Transwell assay showed that cell invasion was remarkably restrained after sh-circ_0001722 or miR-204-5p transfection (Fig. 4c). These findings demonstrated that miR-204-5p contributed to proliferation and invasion of OS cells, and sh-circ_0001722 reduced tumorigenesis in OS cells by directly binding and upregulating miR-204-5p.

### Sh-circ_0001722 mediates OS cells suppression through the miR-204-5p/RUNX2 axis

**Fig. 5:**
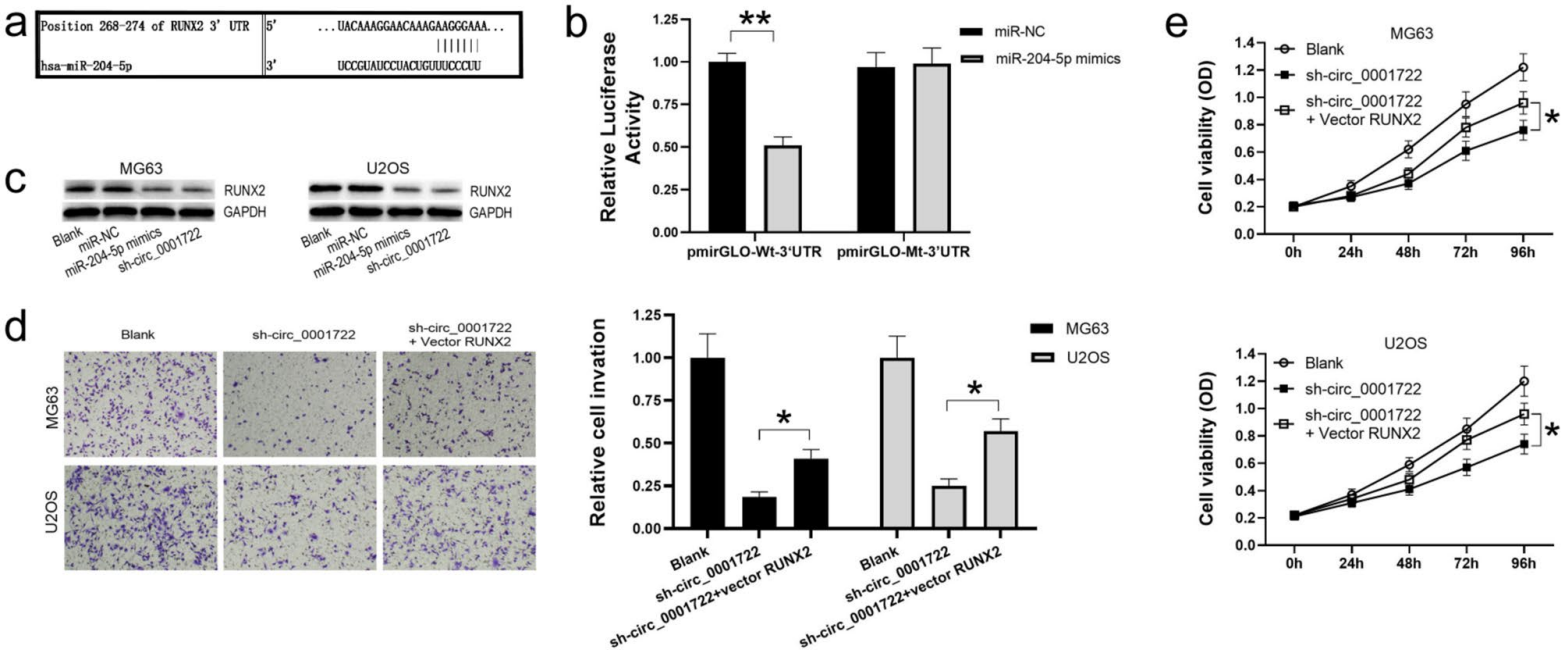
sh-circ_0001722 suppresses proliferation and invasion of human OS cells through miR-204-5p/RUNX2 axis. **a** Computational algorithms predicts that miR-204-5p targets RUNX2 mRNA 3’UTR. **b** Comparing with miR-NC, miR-204-5p can significantly reduce the luciferase activity of pmirGLO-Wt-3’UTR clone in HEK293T cells. However, neither miR-NC nor miR-204-5p can affect the luciferase activity of pmirGLO-Mt-3’UTR clone. **c** Both miR-204-5p and sh-circ_0001722 can suppress RUNX2 expression in human OS cells. **d** Suppression affect by downregulating circ_0001722 on cell proliferation can be rescued by exogenous RUNX2. **e** Suppression affect by downregulating circ_0001722 on cell invasion can be rescued by exogenous RUNX2. NC: negative control. Wt: wild type. Mt: mutant type. *P < 0.05. **P < 0.01

Computational algorithms predicted that miR-204-5p could specifically bind to complementary sequence (268–274 nt) of RUNX2 3’UTR (Fig. 5a). We carried out the 3’UTR luciferase reporter assay to verify whether miR-204-5p can bind to RUNX2 3’UTR and inhibit its expression. The result showed that miR-204-5p could significantly reduce the luciferase activity of pmirGLO-Wt-3’UTR clone, but could not affect the luciferase activity of pmirGLO-Mt-3’UTR clone (Fig. 5b), indicating that miR-204-5p can directly target the 3’UTR of RUNX2 mRNA, leading to the inhibition of its translation. In addition, Western blotting analysis showed that, both miR-204-5p and sh-circ_0001722 could induce a significantly reduction of endogenous RUNX2 expression in OS cells (Fig. 5c). Moreover, rescue assays showed that the cell proliferation ability inhibited by sh-circ_0001722 could be rescued by exogenous RUNX2 (Fig. 5d). The inhibitory effect of sh-circ_0001722 on cell invasion could also be rescued by exogenous RUNX2 as well (Fig. 5e). Our results verified that miR-204-5p contributed to proliferation and invasion of OS cells through targeting RUNX2 mRNA, and sh-circ_0001722 reduced tumorigenesis of OS cells by directly binding and upregulating miR-204-5p/RUNX2 axis.

## Discussion

Our study provides the first evidence that circ_001722 contributes to the malignant progression of OS. CircRNAs are widely accepted to be an unorthodox RNA species generated by alternative splicing of pre-mRNAs (Jia et al. 2019). There are three main classes of circRNAs: exonic circRNAs, exon–intron circRNAs and intronic circRNAs (Ruan et al. 2020). Herein, we revealed upregulated expression of circ_001722 in OS tissues and cells using high-throughput sequencing and qRT-PCR. Functional analyses further validated the role of circ_001722 in promoting the proliferation and metastasis of OS cells both in vivo and in vitro.

The subcellular distribution of RNAs is intimately tied to their biological functions (Buxbaum et al. 2015). Accumulating evidence shows that cytoplasmic circRNAs sponge miRNAs, which represses the translation or induces the degradation of the target mRNAs. Herein, through bioinformatical analysis, we discovered that complementary pairing sites on (82–89 nt) circ_0001722 that can bind to miR-204-5p. Despite this new finding, the involvement of miR-204-5p in the pathogenesis of multiple tumors is not a new phenomenon. MiR-204-5p, derived from TRPM3 Intron 6, plays a key role in many important physiological and pathological processes, especially in the occurrence and development of tumors (Yang et al. 2023). Related studies in prostate cancer, hepatocellular carcinoma, and papillary thyroid cancer have shown that miR-204-5p has a proapoptotic effect (Liu et al. 2015; Lin et al. 2017; Jiang et al. 2016). Zhou Xuefeng et al. pointed out that the overexpression of miR-204-5p in Osteosarcoma cell lines shows similar cell activity damage in vitro and in vivo, further proving the role of miR-204-5p in inhibiting the growth of cancer cells. In addition, when miR-204-5p is overexpressed in Saos-2 and MG63 cells, apoptosis is significantly increased, which indicates that the presence of miR-204-5p induces Programmed cell death in Osteosarcoma (Li et al. 2019). The regulatory effect of CircRNAs on miR-204-5p has been reported in various malignant tumors, such as gastric cancer (Fang et al. 2020; Xu et al. 2021), cholangiocarcinoma (Tu et al. 2021), breast cacer (Bian and Circular 2019), oral squamous cell carcinoma (Tan et al. 2020) and renal cell carcinoma (Wang and Lin 2022). However, reports on the interactions between miR-204-5p and circRNAs in OS are scarce. Herein, we found that miR-204-5p expression was inversely correlated with circ_001722. Functional rescue experiments further revealed that the miR-204-5p inhibitor substantially reversed the suppressive effects of circ_001722 depletion on proliferation and metastasis of OS cells, whereas miR-204-5p could abolish the promotive effects of circ_001722 overexpression.

Evidence indicates that RUNX2 is implicated in diverse biological processes, including bone development, tumor invasion and metastasis (Komori 2020; Zhao et al. 2021). Many studies have shown that osteoblast activity is closely related to the tumorigenesis of OS, and RUNX2 is a recognized transcription factor for osteoblast differentiation during bone development (Li et al. 2015b). Maria Zielenska et al. found RUNX2 was significantly overexpressed in human Osteosarcoma tissue, and the overexpression was significantly associated with poor chemotherapy response in Osteosarcoma (Sadikovic et al. 2010). The reduced expression of RUNX2 protein is associated with up-regulated miRNA in human Osteosarcoma cells (Li et al. 2015b). Overexpression of miR-433 can reduce the level of Runx2, indicating a direct relationship between miR-433 and Runx2 (Li et al. 2008). MiR-155 not only altered mRNA expression in MEF and C2C12 cells, but also downregulated the expression of Runx2 protein (Liu et al. 2018). MiR-204 and miR-211 affect osteoblast differentiation by significantly reducing the expression of Runx2 protein (Huang et al. 2010). Similarly, by directly targeting, miR-590 can also inhibit the expression of Runx2 mRNA (Rohini et al. 2018). In addition, miRNA can indirectly regulate Runx2 by influencing some corepressors and activators (Narayanan et al. 2019). Consistent with previous research, we found that RUNX2 is a downstream target of miR-204-5p in OS cells. Moreover, functional experiments revealed that circ_001722 upregulated RUNX2 expression by sponging miR-204-5p. The network of circRNA-miRNA-mRNA has been a hot research topic in recent years, playing an important role in osteoblast differentiation and other pathways related to osteogenesis (Mohanapriya et al. 2022). For example, CircRNA-23525 can sponge miR-30a-3p, upregulating the expression of RUNX2 in ASCDs, and then promoting osteoblast differentiation (Farshdousti Hagh et al. 2015). CircSIPA1L1 upregulates ALP expression and promotes osteoblast differentiation in SCAPs by sponging miR-204-5p (Wu et al. 2016). CircRNA124534 sponged miR-496 and caused upregulation of b-catenin, which dependent Wnt pathway, further promoted the differentiation of osteoblasts in hDPSCs (Ji et al. 2020). Many other studies also have shown that circRNA-miRNA-mRNA network plays an important role in regulating the expression of ligands, downstream effectors, and receptors, thereby regulating the development of osteoblasts (Mohanapriya et al. 2022). Our results demonstrate once again that circRNA-miRNA-mRNA network plays an important role in the proliferation and invasion of Osteosarcoma cells. But, RUNX2 triggered which pathway activation to accelerate OS progression via mechanisms including suppression of apoptosis and promotion of cell proliferation, migration and invasion, still need to explore. Initial findings indicate the carcinogenesis mediated by the circ_001722/miR-204-5p/RUNX2 axis in OS. In the near future, we will continue to explore the mechanism of circ_001722/miR-204-5p/RUNX2 axis in Osteosarcoma chemotherapy resistance and patient prognosis, and try to find targets for treatment of osteosarcoma and resistance to drug resistance.

## Conclusions

In summary, our research firstly showed that circ_001722 promotes the progression and metastasis of OS via the circ_001722/miR-204-5p/RUNX2 axis. Our findings elucidate a novel regulatory network that may offer new insight into the identification of potential biomarkers or therapeutic targets for OS.

## Supplementary Information

Below is the link to the electronic supplementary material.

Supplementary file1 (TIF 10053 KB)

Supplementary file2 (TIF 16356 KB)

Supplementary file3 (DOCX 14 KB)

Supplementary file4 (DOCX 13 KB)

## Data Availability

All data are fully available without restrictions.
