# Supplementary figures and images for: A novel CircRNA Circ_0001722 regulates proliferation and invasion of osteosarcoma cells through targeting miR-204-5p/RUNX2 axis
Source: J Cancer Res Clin Oncol. 2023 Jul 15;149(14):12779–90. doi: 10.1007/s00432-023-05166-3 (PMC10587032; doi:10.1007/s00432-023-05166-3)

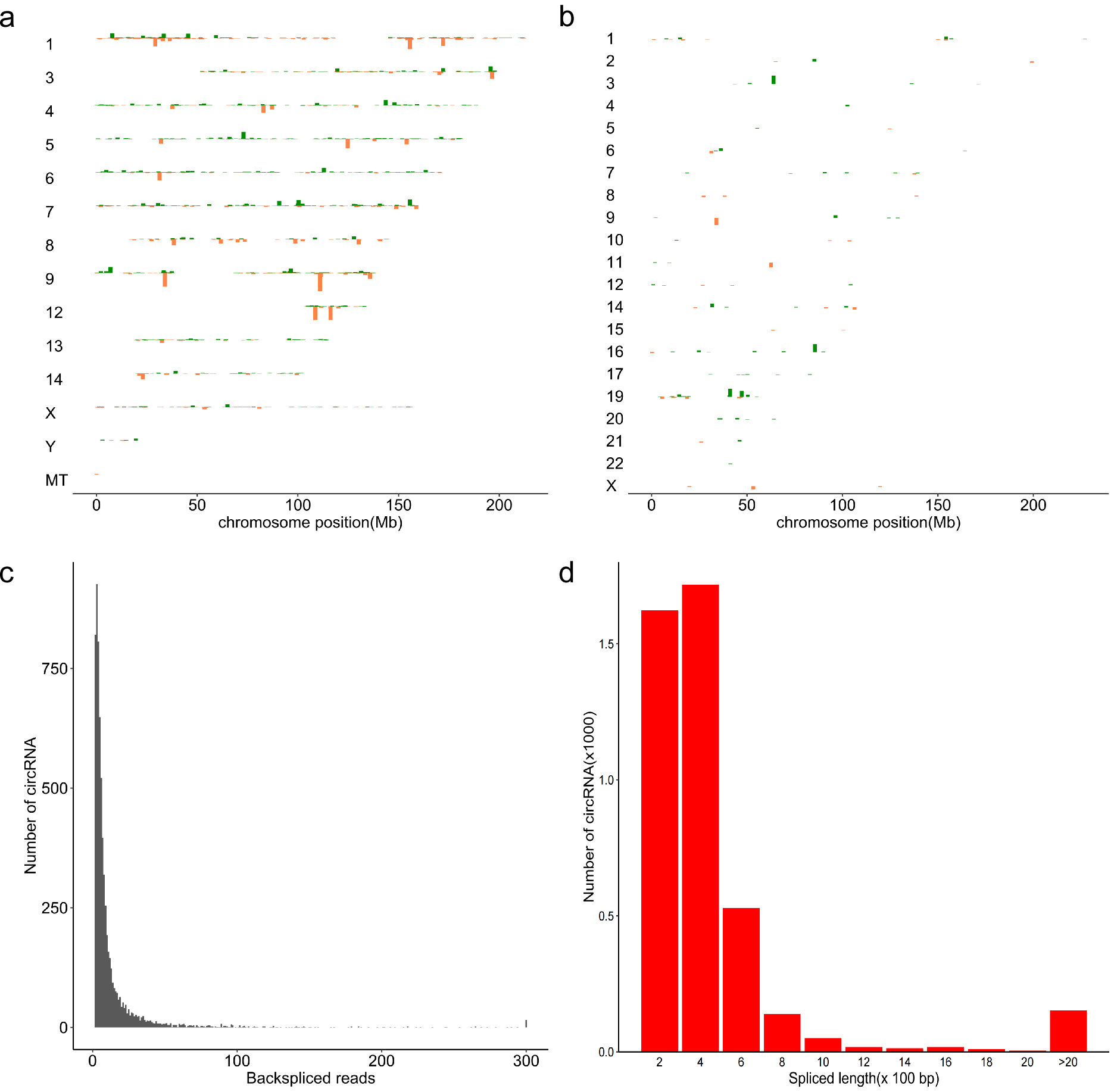

Supplement: Supplementary file 1 — Supplementary file1 (TIF 10053 KB) [file 432_2023_5166_MOESM1_ESM.tif]

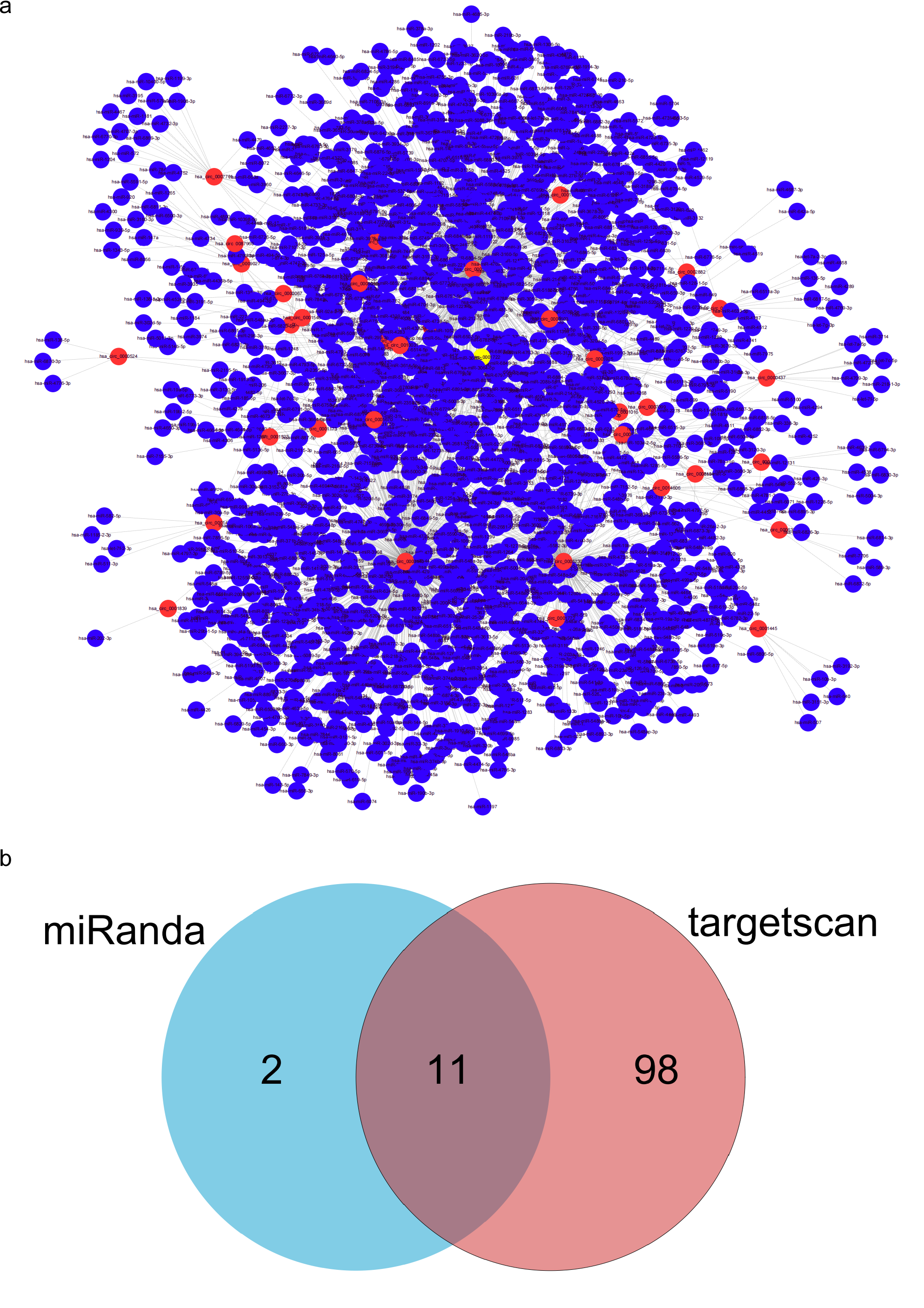

Supplement: Supplementary file 2 — Supplementary file2 (TIF 16356 KB) [file 432_2023_5166_MOESM2_ESM.tif]
