# Supplementary material for: A novel CircRNA Circ_0001722 regulates proliferation and invasion of osteosarcoma cells through targeting miR-204-5p/RUNX2 axis
Source: J Cancer Res Clin Oncol. 2023 Jul 15;149(14):12779–90. doi: 10.1007/s00432-023-05166-3 (PMC10587032; doi:10.1007/s00432-023-05166-3)
Supplement: Supplementary file 3 — Supplementary file3 (DOCX 14 KB) [file 432_2023_5166_MOESM3_ESM.docx]

Supplemental Table 1. The clinicopathological characteristics of OS patients

| **Characteristic** | **Case n (%)** |
| --- | --- |
| **Gender** |  |
| Male | 12(60) |
| Female | 8(40) |
| **Age/year** |  |
| ＜18 | 14(70) |
| ≥18 | 6(30) |
| **Clinical stage** |  |
| I | 1 (5) |
| II | 14(70) |
| III | 5(25) |
| **Tumor size** |  |
| ＜8cm | 5(25) |
| ≥8cm | 15(75) |
| **Location** |  |
| Tibia | 7(35) |
| Femur | 11(55) |
| other | 2(10) |
| **Subtype** |  |
| Osteoblastic | 8(40) |
| Chondroblastic | 7(35) |
| Fibroblastic | 3(15) |
| Mixed | 2(10) |
