# Supplementary material for: A novel CircRNA Circ_0001722 regulates proliferation and invasion of osteosarcoma cells through targeting miR-204-5p/RUNX2 axis
Source: J Cancer Res Clin Oncol. 2023 Jul 15;149(14):12779–90. doi: 10.1007/s00432-023-05166-3 (PMC10587032; doi:10.1007/s00432-023-05166-3)
Supplement: Supplementary file 4 — Supplementary file4 (DOCX 13 KB) [file 432_2023_5166_MOESM4_ESM.docx]

Supplemental Table 2. The primers used in this study

| Name | | Sequence |
| --- | --- | --- |
| circ_0001722 | Forward | TAGCTCTGAAGGTGATCAGGC |
|  | Reverse | CCTTCCCCTAGTTTTTCCAGC |
| miR-204-5p | Forward | ACACTCCAGCTGGGTTCCCTTTGTCATCCTAT |
|  | Reverse | CTCAACTGGTGTCGTGGA |
| GAPDH | Forward | AACGTGTCAGTGGTGGACCTG |
|  | Reverse | AGTGGGTGTCGCTGTTGAAGT |
| U6 | Forward | GCTTCGGCAGCACATATACTAAAAT |
|  | Reverse | CGCTTCACGAATTTGCGTGTCAT |
